# Genome Sequence of a Virulent *Pseudomonas aeruginosa* Strain, 12-4-4(59), Isolated from the Blood Culture of a Burn Patient

**DOI:** 10.1128/genomeA.00079-16

**Published:** 2016-03-03

**Authors:** S. L. Rajasekhar Karna, Tsute Chen, Ping Chen, Trent J. Peacock, Johnathan J. Abercrombie, Kai P. Leung

**Affiliations:** a Dental and Craniofacial Trauma Research and Tissue Regeneration, U.S. Army Institute of Surgical Research, JBSA Fort Sam Houston, Texas, USA; b The Forsyth Institute, Cambridge, Massachusetts, USA

## Abstract

*Pseudomonas aeruginosa* is an opportunistic pathogen that frequently infects wounds, significantly impairs wound healing, and causes morbidity and mortality in burn patients. Here, we report the genome sequence of a virulent strain of *P. aeruginosa*, 12-4-4(59), isolated from the blood culture of a burn patient.

## GENOME ANNOUNCEMENT

*Pseudomonas aeruginosa*, a highly motile, Gram-negative bacterium, is the primary cause of morbidity in clinical burn wound sepsis (1–5). This organism also causes lethal septicemia with its virulence and invasive nature that enables the dissemination from the surface of the burned skin into deeper layers via hair follicles and lymphatic channels, resulting a fatal burn wound sepsis (3, 6, 7).

Strain 12-4-4(59) was isolated from the blood culture of a deceased burn patient with septicemia following second and third degree burn trauma over 70 per cent of her body (8). Rat skin burn wounds infected with 12-4-4(59) had high rates (95.5 per cent) of mortality (3, 8). This strain was extensively used previously at the U.S. Army, Institute of Surgical Research (Fort Sam Houston, Texas) as an infective pathogen in a rat model of burn infection, for development of therapeutic agents to prevent burn wound sepsis. These studies led to the highly successful clinical use of Sulfamylon cream as an effective topical treatment to prevent septicemia in burn patients (3, 8, 9).

To determine the mechanisms underlying the virulent nature of the 12-4-4(59) isolate, the genome was sequenced. *De novo* genome sequencing service was provided by BGI Tech Solutions Co., Ltd. (Cambridge, MA, USA) using a hybrid sequencing approach on two sequencing platforms—the Illumina HiSeq 4000 and the PacBio RS II systems. This approach generated a high-quality close-circular final assembly of 6,431,911 bp in size. The sequence was annotated by the NCBI Prokaryotic Genomes Automatic Annotation Pipeline (PGAP) (10), which generated the following: 5,912 genes, 5,802 coding sequences (CDS), 35 pseudogenes, 12 rRNAs including 4 copies of 5S, 16S, and 23S rRNA each, 35 tRNAs, and 1 noncoding RNA (ncRNA) The genome sequence presented here will be a valuable resource to better understand pathogenome evolution of *P. aeruginosa* 12-4-4-(59) by comparing this pathogenic isolate to the extant genotypes. Moreover, availability of multiple genomes of *P. aeruginosa* will aid in the development of a higher-resolution framework for deciphering the virulence mechanisms and pathophysiology of *P. aeruginosa.*

### Nucleotide sequence accession number.

This genome sequence is deposited in GenBank under the accession number CP013696.
